# Unmet supportive care needs among head and neck cancer survivors in China: multicenter cross-sectional study

**DOI:** 10.3389/fpubh.2026.1776834

**Published:** 2026-05-25

**Authors:** Jing Xie, Ying Huang, Qian Liang, Maojie Zhang, Xialu Liu, Li Ding, Rui Peng, Bijiang Chen, Yan Zuo, Anjie Ren, Meifang Xu, Rong Yu

**Affiliations:** 1 Outpatient Blood Collection Center, West China Hospital, Sichuan University/West China School of Nursing, Sichuan University, Chengdu, China; 2 Department of Otolaryngology-Head and Neck Surgery, West China Hospital, Sichuan University/West China School of Nursing, Sichuan University, Chengdu, China; 3 West China Hospital of Stomatology, Sichuan University, Chengdu, China; 4 Intensive Care Unit, West China Hospital, Sichuan University, Chengdu, China; 5 Information Department, Guangzhou Fuda Cancer Hospital, Guangzhou, China; 6 Department of Gynecology and Obstetrics Nursing, West China Second University Hospital, Sichuan University, Chengdu, China; 7 West China School of Nursing, Sichuan University, Chengdu, China; 8 Key Laboratory of Birth Defects and Related Disease of Women and Children (Sichuan University), Ministry of Education, Chengdu, China; 9 Healthcare and Education Research Center, Chengdu Gongyun Education and Management Research Institute, Chengdu, China; 10 Shulan (Hangzhou) Hospital, Hangzhou, China

**Keywords:** head and neck cancer, nasopharyngeal carcinoma, quality of life, SCNS-SF34, supportive care needs, survivorship

## Abstract

**Background:**

Survival after head and neck cancer treatment has improved, shifting attention toward survivorship quality and post-treatment service gaps. Evidence from China that quantifies unmet supportive care needs using standardized instruments across multiple regions remains limited.

**Methods:**

We conducted a multicenter cross-sectional study in three tertiary hospitals in East, Middle, and Southwest China. Adults with histologically confirmed head and neck cancer were recruited during routine follow-up 3 to 24 months after curative-intent treatment. Supportive care needs were assessed with the Supportive Care Needs Survey Short Form (SCNS-SF34) and the head and neck module (SCNS-HNC), and head and neck-related quality of life was measured with University of Washington Quality of Life version 4 (UW-QOL v4). The primary outcome was any moderate-to-high unmet need on SCNS-SF34. We reported overall and hospital-specific prevalence, domain-level burden, and correlates from mixed-effects logistic regression with hospital clustering interpreted cautiously because only three centers were included.

**Results:**

Among 1,407 surveyed survivors, 1,239 questionnaires were valid for analysis (88.1%). Overall, 78.2% reported at least one moderate-to-high unmet need. Health system and information (34.2%) and psychological needs (31.2%) were the most prevalent SCNS-SF34 domains. Dry mouth or sticky saliva (54.6%) and swallowing difficulties (36.8%) were the leading head and neck-specific unmet needs. The median number of moderate-to-high SCNS-SF34 needs per patient was 3 (IQR 2 to 5). Lower UW-QOL composite score (per 10-point decrease: aOR 1.61), rural residence (aOR 1.25), monthly household income below 5,000 CNY (aOR 1.45), stage III to IV disease (aOR 1.25), and multimorbidity of at least two comorbidities (aOR 1.83) were associated with higher odds of unmet needs. In a hospital fixed-effect sensitivity model, the direction and magnitude of the main patient-level associations were materially unchanged.

**Conclusion:**

Unmet supportive care needs were common among Chinese head and neck cancer survivors in early survivorship, particularly in information, psychological, salivary, and swallowing domains. The observed associations suggest that routine needs screening and multidisciplinary follow-up pathways may help identify survivors who are more likely to report substantial unmet needs, especially in socioeconomically vulnerable groups. Any service-level interpretation should remain cautious because the evidence is observational and based on three tertiary centers.

## 1 Introduction

Survival after head and neck cancer treatment has improved with advances in radiotherapy delivery, multimodal treatment, imaging, and supportive management, shifting clinical attention toward survivorship quality and the organization of post-treatment services (1–3). For many head and neck cancers, the first 2 years after treatment represent a high-need period characterized by persistent symptoms, functional limitations, and psychosocial distress that may not be fully addressed within conventional follow-up models (4–6). Survivorship planning carries particular salience because care pathways often concentrate in tertiary centers, patient catchment areas are large, and rehabilitation and psychosocial resources vary across regions and institutions (7–9). In the current study, head and neck cancer (HNC) refers to malignancies managed within ENT and head and neck oncology services, including nasopharyngeal carcinoma and other major head and neck sites represented in the cohort.

Nasopharyngeal carcinoma contributes substantially to the ENT oncology workload in many Chinese tertiary hospitals, and its dominant treatment modality, radiotherapy with or without chemotherapy, is associated with a distinctive profile of late and subacute effects (10–12). Xerostomia and altered saliva consistency, dysgeusia, dysphagia, hearing-related problems, fatigue, sleep disruption, and neck or shoulder limitations can persist well beyond treatment completion, influencing nutrition, communication, return to work, social participation, and mental health (13–15). Even when disease control is achieved, the lived burden of survivorship can remain high, and the nature of supportive care required extends beyond symptom control to include education, navigation, rehabilitation, psychosocial support, and access to multidisciplinary services (12, 16).

Supportive care needs are clinically important because they represent the patient patients perspective on gaps between experienced problems and received assistance (17, 18). Unlike symptom severity alone, needs assessments capture a service-oriented construct that directly informs survivorship program design, referral pathways, and resource allocation (17, 19). A needs-based approach is also well aligned with public health priorities because it supports equity-oriented planning, identifies vulnerable subgroups, and enables benchmarking across institutions (17, 20, 21). In practice, systematic needs assessment remains inconsistently implemented in routine HNC follow-up, and many centers rely on clinician-led surveillance focused on recurrence detection, which can leave rehabilitation and psychosocial concerns under-recognized (1, 22–24).

Existing head and neck survivorship studies have frequently been single-center, have mixed treatment phases, or have prioritized symptom and quality-of-life outcomes without explicitly quantifying unmet supportive care needs (25–27). Evidence from China that integrates standardized supportive care needs instruments with a head and neck-specific quality-of-life measure across multiple regions remains limited (28, 29). Regional heterogeneity matters because differences in socioeconomic context, referral systems, insurance coverage, travel burden, and availability of speech and swallowing therapy, dental services, nutrition counseling, and psychosocial care can plausibly shape both the magnitude and the pattern of unmet needs (19, 25, 26, 30). A multicenter approach across distinct Chinese regions can therefore provide more generalizable estimates and offer actionable comparisons that can guide service improvement.

The specific contribution of the present study is the combination of a three-region multicenter design, an early-survivorship NPC-heavy cohort, concurrent use of SCNS-SF34 and SCNS-HNC with UW-QOL v4, and hospital-clustered multivariable analysis under a harmonized protocol. Relative to prior Chinese survivorship studies, novelty lies not in examining need burden alone but in integrating standardized supportive-care and functional instruments across multiple tertiary settings while comparing crude and adjusted patterns across hospitals.

To address this gap, we conducted a multicenter cross-sectional study to estimate the prevalence of moderate-to-high unmet supportive care needs and to describe domain-level and head and neck-specific need patterns in an NPC-heavy survivorship cohort. Secondary objectives were to characterize functional profiles on UW-QOL, to evaluate associations between functional impairment and unmet needs, and to examine crude and risk-adjusted differences in unmet needs across hospitals as a pragmatic indicator of service variation.

We hypothesized that unmet supportive care needs would be common during early survivorship and would be most prominent in domains related to health system and information and psychological concerns, alongside head and neck-specific needs linked to xerostomia-related problems and swallowing. We further hypothesized that poorer UW-QOL composite scores, socioeconomic disadvantage, and greater clinical burden would be associated with higher odds of reporting moderate-to-high unmet needs, and that hospital-level differences would persist after accounting for measured patient characteristics, suggesting that survivorship service configuration and regional context contribute meaningfully to needs burden.

## 2 Methods

### 2.1 Study design and setting

We conducted a multicenter cross-sectional study of head and neck cancer survivors attending routine post-treatment follow-up clinics. The design aimed to estimate the prevalence and distribution of moderate-to-high unmet supportive care needs and to identify patient-level correlates while accounting for between-hospital clustering.

Participants were recruited from three tertiary hospitals located in three Chinese regions, including East China (Hospital E), Middle China (Hospital M), and Southwest China (Hospital S). Each hospital maintains dedicated ENT oncology services and structured survivorship follow-up pathways, including high-volume nasopharyngeal carcinoma care supported by radiotherapy and chemoradiotherapy programs. Recruitment occurred over a three-month period using harmonized procedures and a single protocol across all sites.

Because recruitment occurred in tertiary follow-up clinics, the sampled population was intended to represent survivors who remained engaged with specialist post-treatment care rather than all survivors in the community. We therefore interpreted prevalence estimates as clinic-based early survivorship estimates and considered possible underrepresentation of survivors with access barriers, follow-up outside tertiary centers, or poorer post-treatment retention.

### 2.2 Participants, eligibility, and recruitment

Eligible participants were adults aged 18 years or older with histologically confirmed head and neck cancer managed within ENT and head and neck oncology services who had completed curative-intent primary treatment and were within a prespecified survivorship window of 3 to 24 months from treatment completion. Participants were required to be clinically stable at the time of survey, able to provide informed consent, and able to complete Mandarin-language questionnaires either independently or with standardized interviewer assistance.

We excluded patients with documented recurrence or progression or those receiving active salvage therapy at the time of survey, because supportive care needs during active recurrent disease differ conceptually and clinically from survivorship needs in the early post-treatment window (31). We also excluded patients who could not complete the survey due to severe cognitive impairment or acute psychiatric instability, and questionnaires that failed prespecified validity criteria due to excessive missingness or internal inconsistency (32–34).

At each hospital, consecutive sampling was used among eligible survivors presenting for follow-up visits during the recruitment window. Trained research staff approached patients in clinic waiting areas or immediately before consultation, confirmed eligibility, obtained written informed consent, and administered the survey packet using Wenjuanxing, an electronic survey system widely employed by Chinese scholars. Clinical variables were abstracted from the medical records.

Interviewer assistance was permitted when participants reported difficulty reading, typing, or sustaining attention because of fatigue or treatment-related functional problems. Across the three hospitals, interviewer-assisted completion was documented in approximately 14% of questionnaires, with no major between-hospital imbalance on routine quality-control review. In that setting, research staff read items verbatim, recorded the participant’s selected response without interpretation, and documented assisted administration within site logs.

### 2.3 Measures

#### 2.3.1 Primary outcome: supportive care needs

Supportive care needs were assessed using the Supportive Care Needs Survey Short Form (SCNS-SF34) together with the head and neck cancer-specific module (SCNS-HNC). The SCNS-SF34 captures perceived needs over the prior month across five domains: health system and information, psychological, physical and daily living, patient care and support, and sexuality (17, 19, 28, 35). Items use a five-point need scale anchored from no need or satisfied to low need, moderate need, and high need. The SCNS-HNC module complements the core survey by assessing head and neck-specific needs that are common in ENT survivorship, including concerns related to xerostomia and sticky saliva, swallowing and chewing, speech and voice, hearing, nutrition information, oral care, neck and shoulder function, and lifestyle counseling (19, 23).

Mandarin Chinese versions of SCNS-SF34, SCNS-HNC, and UW-QOL v4 that had been used in prior oncology research were administered uniformly across sites. Internal consistency in the present analytic sample was acceptable to high, with Cronbach’s alpha values of 0.94 for SCNS-SF34, 0.91 for SCNS-HNC, and 0.89 for UW-QOL v4 composite-domain scoring. The study emphasis was on standardized application of established instruments rather than *de novo* scale development, so questionnaire quality assurance focused on protocol harmonization, completeness checks, and adherence to published scoring rules.

The primary endpoint was a patient-level binary indicator of any moderate-to-high unmet supportive care need on the SCNS-SF34, defined as at least one SCNS-SF34 item rated as moderate or high need. In addition to the primary endpoint, we prespecified domain-level prevalence for each SCNS-SF34 domain defined as at least one moderate-to-high need item within that domain, and we computed continuous domain burden scores using standardized 0–100 transformations, with higher scores indicating greater need burden. For the SCNS-HNC module, we reported item-level prevalence of moderate-to-high unmet need to characterize head and neck-specific survivorship needs in an NPC-heavy cohort across regions.

We selected the person-level definition of any moderate-to-high SCNS-SF34 item as the primary endpoint because it is sensitive for clinical screening and identifies survivors with at least one materially unmet concern that may warrant follow-up. Because this definition can yield higher patient-level prevalence than stricter domain-based rules, we also reported domain-level prevalence, continuous domain burden scores, and the number of moderate-to-high SCNS-SF34 needs per patient to provide complementary information on burden.

#### 2.3.2 Head and neck quality of life (explanatory secondary outcome)

Head and neck-related quality of life was assessed using the University of Washington Quality of Life questionnaire version 4 (UW-QOL v4), which measures function and symptoms over the prior 7 days across 12 domains: pain, appearance, activity, recreation, swallowing, chewing, speech, shoulder, taste, saliva, mood, and anxiety (36–38). Each domain is scored from 0 to 100, with higher scores indicating better function or less symptom burden. We defined the UW-QOL composite score *a priori* as the mean of the 12 domain scores and used this composite as the principal explanatory variable capturing overall head and neck functional status in survivorship, while also reporting domain-level scores to identify the most affected functional areas in this cohort.

UW-QOL v4 was included as an indicator of overall functional status rather than as a causal determinant of unmet need. We recognized partial conceptual overlap between quality of life and supportive care needs and interpreted the UW-QOL coefficient as reflecting broader survivorship burden rather than a wholly distinct construct.

#### 2.3.3 Covariates

Covariates reflected a conceptual framework linking supportive care needs to sociodemographic vulnerability, clinical burden, and functional impairment. Sociodemographic variables included age, sex, residence (urban or rural), education, monthly household income, and insurance type. Clinical variables included primary cancer site (nasopharyngeal carcinoma versus other head and neck sites), stage at diagnosis, primary treatment modality, time since treatment completion within the 3 to 24 month window, comorbidity count, and the presence of feeding tube or tracheostomy at the time of survey. Sociodemographic data were obtained by self-report and clinical variables were extracted from records using the standardized abstraction form.

### 2.4 Data quality assurance and validity rules

All sites operated under a common quality assurance SOP. Site staff completed centralized training on recruitment, consent, survey administration, and chart abstraction procedures. Surveys were checked for completeness at the point of administration, and participants were invited to complete missed items when clinically appropriate. Data were entered with range and logic checks, with double-entry verification for paper sources or dual review for electronic capture. The coordinating team conducted routine central monitoring to identify out-of-range values, implausible combinations, and site-specific patterns of missingness.

A questionnaire was considered valid for analysis if the participant met eligibility criteria including the 3 to 24 month survivorship window and if the primary outcome instruments met prespecified completeness thresholds without evidence of systematic response errors. Questionnaires with excessive missingness in SCNS-SF34 or UW-QOL v4 or with internal inconsistencies that rendered the primary endpoint non-derivable were classified as non-valid. These rules were applied uniformly across the three hospitals before analysis.

No imputation was used for the primary endpoint. Participants with non-derivable primary outcome data or questionnaires failing validity checks were excluded before analysis, and regression models were fit on complete cases for prespecified covariates.

### 2.5 Statistical analysis

All analyses were conducted using two-sided tests with an alpha level of 0.05. We summarized categorical variables as counts and percentages and continuous variables as means with standard deviations, using medians with interquartile ranges only if distributions were clearly non-normal. We estimated the overall and hospital-specific prevalence of the primary endpoint with 95% confidence intervals and summarized domain-level prevalence and mean standardized domain scores for SCNS-SF34. For SCNS-HNC, we reported item-level prevalence of moderate-to-high unmet need overall and by hospital.

To evaluate correlates of unmet supportive care needs while accounting for clustering by hospital, we fitted a mixed-effects logistic regression model with a random intercept for hospital. The dependent variable was the primary endpoint of any moderate-to-high unmet need on SCNS-SF34. Fixed-effect covariates included prespecified sociodemographic and clinical variables and the UW-QOL composite score, modeled as a continuous predictor and scaled per 10-point decrease to yield interpretable effect sizes. Model results were reported as adjusted odds ratios with 95% confidence intervals and *p* values. We also quantified between-hospital heterogeneity using the estimated random-intercept variance and a corresponding intraclass correlation as descriptive context.

Covariates were prespecified from the conceptual framework rather than selected by automated stepwise procedures. Multicollinearity was assessed before model fitting, and all variance inflation factors were below 2.5. Model diagnostics included inspection of residual patterns, influence statistics, and calibration of predicted probabilities. Given that only three hospitals were available, the random intercept was used primarily to account for within-hospital correlation when estimating patient-level associations; hospital-level variance components and adjusted between-hospital comparisons were treated as descriptive and interpreted cautiously. A fixed-effect model treating hospital as categorical was fitted as a sensitivity analysis.

Hospital-level adjusted prevalence was estimated by standardizing model-based predicted probabilities to the overall covariate distribution, yielding risk-adjusted prevalence estimates and confidence intervals for each hospital. Descriptive comparisons across hospitals for baseline characteristics and unadjusted outcomes used chi-square tests for categorical variables and ANOVA for continuous variables, but inference regarding correlates and hospital differences emphasized the multilevel model consistent with the multicenter design.

Because the number of clusters was small, hospital differences were described as pragmatic signals of possible service variation rather than definitive estimates of regional or system-level effects.

### 2.6 Ethics

The study was ethically approved by the Ethics Committee of West China Hospital, Sichuan University. All participants provided written informed consent before participation. Data were de-identified prior to central aggregation and analysis, stored on secure servers, and accessed only by authorized study personnel.

## 3 Results

**Figure 1:**
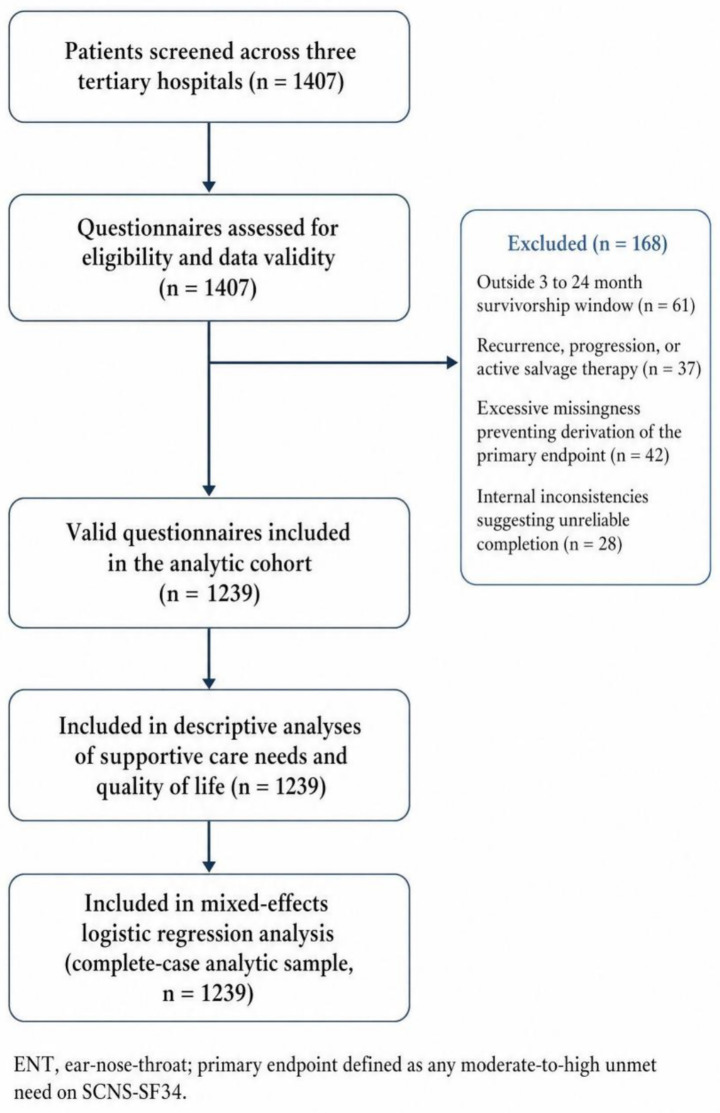
Participant flow diagram for the multicenter study. A total of 1,407 patients were screened across three tertiary hospitals in China. After eligibility assessment and data-validity review, 168 questionnaires were excluded because participants were outside the 3 to 24 month survivorship window, had recurrence, progression, or active salvage therapy, had excessive missingness preventing derivation of the primary endpoint, or had internal inconsistencies suggesting unreliable completion. The final analytic cohort included 1,239 head and neck cancer survivors, all of whom were included in the descriptive analyses and mixed-effects logistic regression. The primary endpoint was any moderate-to-high unmet supportive care need on the SCNS-SF34.

Across the three hospitals, 1,407 patients were surveyed, with 1,239 questionnaires valid for analysis (88.1%). Of the remaining 168 surveys, 61 were excluded because participants were outside the 3 to 24 month survivorship window, 37 because recurrence, progression, or active salvage therapy was documented at screening, 42 because excessive missingness prevented derivation of the primary endpoint, and 28 because internal inconsistencies suggested unreliable instrument completion (Figure 1).

### 3.1 Participant characteristics

The analytic cohort had a mean age of 53.2 years and was predominantly male. Rural residence constituted slightly more than half of the cohort, and nearly half of participants reported monthly household income below 5,000 CNY. NPC represented the majority cancer site across all three hospitals, and most participants had stage III to IV disease at diagnosis, consistent with the NPC-heavy case mix and treatment patterns. The predominant primary treatment was radiotherapy with or without chemotherapy. Time since treatment completion was distributed across the prespecified 3 to 24 month window, enabling characterization of early survivorship needs in a period of ongoing functional recovery and persistent treatment sequelae. Detailed characteristics overall and by hospital are presented in Table 1.

**Table 1:** Participant characteristics overall and by hospital.

Category		East	Middle	Southwest	Overall	*P* value*
		(*n* = 438)	(*n* = 411)	(*n* = 390)	(N = 1,239)	
Age	Years, mean (SD)	52.3 (10.4)	53.3 (10.7)	54.1 (10.9)	53.2 (10.7)	0.055
Sex	Male	315 (71.9)	310 (75.4)	292 (74.9)	917 (74.0)	0.455
	Female	123 (28.1)	101 (24.6)	98 (25.1)	322 (26.0)	
Residence	Urban	229 (52.3)	188 (45.7)	175 (44.9)	592 (47.8)	0.062
	Rural	209 (47.7)	223 (54.3)	215 (55.1)	647 (52.2)	
Education	≤Junior middle	182 (41.6)	196 (47.7)	176 (45.1)	554 (44.7)	0.435
	High school/TVET	142 (32.4)	122 (29.7)	114 (29.2)	378 (30.5)	
	College+	114 (26.0)	93 (22.6)	100 (25.6)	307 (24.8)	
Monthly household Income (CNY)	<5,000	190 (43.4)	212 (51.6)	193 (49.5)	595 (48.0)	0.037
	5,000–9,999	153 (34.9)	140 (34.1)	133 (34.1)	426 (34.4)	
	≥10,000	95 (21.7)	59 (14.4)	64 (16.4)	218 (17.6)	
Insurance	UEBMI	150 (34.2)	115 (28.0)	107 (27.4)	372 (30.0)	0.377
	URBMI	105 (24.0)	103 (25.1)	99 (25.4)	307 (24.8)	
	NRCMS	168 (38.4)	180 (43.8)	172 (44.1)	520 (42.0)	
	Other	15 (3.4)	13 (3.2)	12 (3.1)	40 (3.2)	
Primary cancer site	NPC	250 (57.1)	241 (58.6)	227 (58.2)	718 (58.0)	0.919
	Larynx	55 (12.6)	47 (11.4)	44 (11.3)	146 (11.8)	
	Oral cavity	45 (10.3)	43 (10.5)	34 (8.7)	122 (9.8)	
	Oro/hypopharynx	40 (9.1)	39 (9.5)	32 (8.2)	111 (9.0)	
	Sinonasal	26 (5.9)	21 (5.1)	25 (6.4)	72 (5.8)	
	Thyroid/other	22 (5.0)	20 (4.9)	28 (7.2)	70 (5.6)	
Stage at diagnosis	I	35 (8.0)	29 (7.1)	30 (7.7)	94 (7.6)	0.933
	II	95 (21.7)	85 (20.7)	93 (23.8)	273 (22.0)	
	III	160 (36.5)	150 (36.5)	140 (35.9)	450 (36.3)	
	IV	148 (33.8)	147 (35.8)	127 (32.6)	422 (34.1)	
Primary treatment	RT ± CT	275 (62.8)	285 (69.3)	249 (63.8)	809 (65.3)	0.222
	Surgery+Adjuvant	95 (21.7)	70 (17.0)	85 (21.8)	250 (20.2)	
	Surgery alone	45 (10.3)	32 (7.8)	41 (10.5)	118 (9.5)	
	Other	23 (5.3)	24 (5.8)	15 (3.8)	62 (5.0)	
Time since treatment completion (months)	3–6	90 (20.5)	95 (23.1)	86 (22.1)	271 (21.9)	0.714
	7–12	120 (27.4)	115 (28.0)	111 (28.5)	346 (27.9)	
	13–18	115 (26.3)	100 (24.3)	110 (28.2)	325 (26.2)	
	19–24	113 (25.8)	101 (24.6)	83 (21.3)	297 (24.0)	
Comorbidities (count)	0	250 (57.1)	214 (52.1)	218 (55.9)	682 (55.0)	0.517
	1	129 (29.5)	133 (32.4)	110 (28.2)	372 (30.0)	
	≥2	59 (13.5)	64 (15.6)	62 (15.9)	185 (14.9)	
Feeding tube *in situ*	Yes	28 (6.4)	34 (8.3)	27 (6.9)	89 (7.2)	0.554
	No	410 (93.6)	377 (91.7)	363 (93.1)	1,150 (92.8)	
Tracheostomy in situ	Yes	10 (2.3)	13 (3.2)	14 (3.6)	37 (3.0)	0.527
	No	428 (97.7)	398 (96.8)	376 (96.4)	1,202 (97.0)	

* P values are descriptive comparisons across hospitals (ANOVA for age; chi-square tests for categorical variables).

### 3.2 Prevalence and distribution of unmet supportive care needs on SCNS-SF34

Overall, 969 of 1,239 participants met the prespecified primary endpoint (prevalence, 78.2, 95% CI: 75.8–80.4%). Unadjusted prevalence varied by hospital, with Hospital E in East China showing the lowest prevalence and Hospital S in Southwest China showing the highest prevalence. Domain-level prevalence patterns indicated that needs related to health system and information and psychological concerns were most common, followed by physical and daily living needs, patient care and support needs, and sexuality-related needs. Standardized domain scores showed a similar ranking of burden across domains, with the largest mean scores observed for health system and information and psychological needs (Table 2).

**Table 2:** SCNS-SF34 prevalence and domain scores.

Outcome	East (*n* = 438)	Middle (*n* = 411)	Southwest (*n* = 390)	Overall (*N* = 1,239)
Any moderate-to-high unmet need (SF34; ≥1 item)	323 (73.7)	322 (78.3)	324 (83.1)	969 (78.2)
Health system and information (≥1 item)	127 (29.0)	137 (33.3)	160 (41.0)	424 (34.2)
Psychological (≥1 item)	121 (27.6)	119 (29.0)	147 (37.7)	387 (31.2)
Physical and daily living (≥1 item)	106 (24.2)	130 (31.6)	119 (30.5)	355 (28.7)
Patient care & support (≥1 item)	100 (22.8)	87 (21.2)	102 (26.2)	289 (23.3)
Sexuality (≥1 item)	75 (17.1)	64 (15.6)	64 (16.4)	203 (16.4)
Health system & information score (0–100), mean (SD)	22.7 (15.3)	26.0 (15.9)	28.1 (15.7)	25.5 (15.8)
Psychological score (0–100), mean (SD)	20.4 (14.8)	23.8 (15.0)	25.9 (14.8)	23.3 (15.0)
Physical & daily living score (0–100), mean (SD)	18.3 (13.8)	21.5 (13.8)	24.1 (14.4)	21.2 (14.2)
Patient care & support score (0–100), mean (SD)	17.3 (12.6)	19.7 (13.7)	21.8 (14.7)	19.5 (13.8)
Sexuality score (0–100), mean (SD)	13.9 (11.6)	15.8 (12.8)	16.9 (12.9)	15.5 (12.5)

Among participants meeting the primary endpoint, multidomain needs were common. The median number of moderate-to-high SCNS-SF34 needs per patient was 3 (IQR 2 to 5), indicating that survivorship service planning should anticipate concurrent informational, psychological, and symptom-related needs rather than isolated single-domain concerns.

### 3.3 Head and neck cancer-specific unmet needs on SCNS-HNC

Head and neck cancer-specific unmet needs were frequent and highly patterned in this cohort. The most prevalent moderate-to-high unmet needs related to dry mouth or sticky saliva, swallowing difficulties, and nutrition or diet information, consistent with an NPC-heavy survivorship population treated predominantly with radiotherapy-based regimens. Hearing-related needs were also common, reflecting the late effects profile of head and neck irradiation. Lifestyle counseling needs related to stopping smoking and alcohol were less frequent than symptom- and function-related needs but were not negligible, particularly in the Middle China and Southwest China hospitals. Item-level prevalence overall and by hospital is presented in Table 3.

**Table 3:** SCNS-HNC item-level prevalence of moderate-to-high unmet needs.

SCNS-HNC item (moderate-to-high unmet need)	East (*n* = 438)	Middle (*n* = 411)	Southwest (*n* = 390)	Overall (*N* = 1,239)
Dry mouth/sticky saliva	225 (51.4)	237 (57.7)	214 (54.9)	676 (54.6)
Swallowing difficulties	140 (32.0)	149 (36.3)	167 (42.8)	456 (36.8)
Nutrition/diet information	125 (28.5)	137 (33.3)	131 (33.6)	393 (31.7)
Hearing problems	106 (24.2)	117 (28.5)	115 (29.5)	338 (27.3)
Chewing/eating difficulties	75 (17.1)	82 (20.0)	98 (25.1)	255 (20.6)
Speech/voice problems	68 (15.5)	72 (17.5)	82 (21.0)	222 (17.9)
Oral hygiene/dental care	64 (14.6)	71 (17.3)	81 (20.8)	216 (17.4)
Neck/shoulder stiffness or mobility	60 (13.7)	81 (19.7)	75 (19.2)	216 (17.4)
Weight maintenance/gain	77 (17.6)	71 (17.3)	77 (19.7)	225 (18.2)
Help to stop smoking	42 (9.6)	58 (14.1)	66 (16.9)	166 (13.4)
Help to stop drinking alcohol	40 (9.1)	39 (9.5)	49 (12.6)	128 (10.3)
Stoma/voice prosthesis care	26 (5.9)	25 (6.1)	19 (4.9)	70 (5.6)

### 3.4 UW-QOL v4 scores and functional profile

The overall UW-QOL composite score was 74.0 with a standard deviation of 6.0. Across the 12 UW-QOL domains, the lowest mean scores were observed for saliva and taste, with swallowing also showing substantial impairment, indicating a persistent burden of xerostomia, dysgeusia, and dysphagia within the first 2 years after treatment. Hospital-level differences were apparent, with higher composite scores in the East China hospital and lower scores in the Southwest China hospital, consistent with the parallel pattern observed for unmet needs prevalence (Table 4).

**Table 4:** UW-QOL v4 domain scores (0–100; higher = better).

UW-QOL v4 domain score	East (*n* = 438)	Middle (*n* = 411)	Southwest (*n* = 390)	Overall (*N* = 1,239)
Pain (0–100), mean (SD)	89.0 (12.4)	87.5 (12.9)	87.1 (13.3)	87.9 (12.9)
Appearance (0–100), mean (SD)	77.1 (18.6)	75.0 (17.7)	74.5 (17.5)	75.6 (18.0)
Activity (0–100), mean (SD)	81.3 (15.6)	79.5 (16.1)	79.2 (16.2)	80.0 (16.0)
Recreation (0–100), mean (SD)	78.6 (16.6)	77.5 (16.1)	77.7 (16.1)	77.9 (16.3)
Swallowing (0–100), mean (SD)	71.8 (19.7)	69.1 (20.4)	66.0 (21.8)	69.1 (20.8)
Chewing (0–100), mean (SD)	73.6 (21.5)	70.9 (21.8)	71.6 (20.1)	72.1 (21.2)
Speech (0–100), mean (SD)	80.9 (17.3)	78.1 (18.0)	78.0 (17.7)	79.0 (17.7)
Shoulder (0–100), mean (SD)	80.0 (17.8)	79.4 (17.8)	79.8 (17.6)	79.7 (17.7)
Taste (0–100), mean (SD)	65.4 (22.7)	62.8 (23.8)	62.5 (23.5)	63.6 (23.3)
Saliva (0–100), mean (SD)	57.0 (27.7)	54.2 (25.9)	53.9 (26.3)	55.1 (26.7)
Mood (0–100), mean (SD)	80.3 (17.6)	75.9 (18.3)	75.2 (18.8)	77.2 (18.3)
Anxiety (0–100), mean (SD)	72.4 (20.1)	70.0 (20.4)	69.9 (20.5)	70.8 (20.4)
UW-QOL composite (mean of 12 domains), mean (SD)	75.6 (5.9)	73.3 (5.8)	72.9 (6.0)	74.0 (6.0)

### 3.5 Multilevel correlates of unmet supportive care needs

In the mixed-effects logistic regression with hospital modeled as a random intercept, lower UW-QOL composite scores were strongly associated with higher odds of meeting the primary unmet-needs endpoint. Socioeconomic disadvantage and clinical burden also showed independent associations, including lower household income, rural residence, advanced stage at diagnosis, and multimorbidity. Higher education and being further from treatment completion within the survivorship window were associated with reduced odds of unmet needs, consistent with recovery trajectories and improved navigation of survivorship resources over time. Treatment modality was also associated with unmet needs, with surgery-based treatment showing lower odds than radiotherapy with or without chemotherapy, reflecting the NPC-heavy radiotherapy cohort and the persistence of xerostomia and swallowing-related effects. In a sensitivity analysis specifying hospital as a fixed effect, the principal patient-level coefficients remained directionally consistent, supporting the robustness of the main inferences.

Age also showed a statistically significant but modest association, with an adjusted OR of 1.09 per 10-year increase, indicating a small effect size at the individual level despite precise estimation in this large sample. By contrast, NPC primary site did not retain conventional statistical significance after adjustment, which suggests that treatment-related functional burden may have been more informative than tumor site alone within this cohort (Table 5).

**Table 5:** Mixed-effects logistic regression for any moderate-to-high unmet need (SCNS-SF34).

Predictor	Reference	Adjusted OR	95% CI	*P* value
Age (per 10-year increase)		1.09	1.07–1.12	<0.001
Male sex	Female	0.97	0.83–1.14	0.715
Rural residence	Urban	1.25	1.02–1.52	0.028
Education: College+	≤High school/TVET	0.66	0.51–0.85	0.002
Monthly income <5,000 CNY	≥5,000 CNY	1.45	1.17–1.79	0.001
Time since treatment: 7–12 months	3–6 months	0.83	0.63–1.09	0.177
Time since treatment: 13–24 months	3–6 months	0.65	0.54–0.79	<0.001
Stage III-IV	Stage I-II	1.25	1.06–1.48	0.009
Comorbidities ≥2	0–1	1.83	1.19–2.83	0.006
NPC primary site	Other HNC sites	1.18	0.98–1.42	0.088
Treatment: surgery-based	RT ± CT	0.72	0.57–0.92	0.008
Treatment: other	RT ± CT	0.64	0.36–1.12	0.117
UW-QOL composite (per 10-point decrease)		1.61	1.52–1.70	<0.001

The estimated hospital random-intercept variance indicated modest but non-negligible between-hospital heterogeneity after adjustment, consistent with the conceptual importance of regional service configuration and survivorship program capacity. The small number of clusters limited precision for variance components, but the direction and magnitude were consistent with the observed hospital-level differences in crude prevalence.

### 3.6 Crude and risk-adjusted hospital prevalence of unmet needs

Crude prevalence of the primary unmet-needs endpoint ranged from 73.7% in the East China hospital to 83.1% in the Southwest China hospital. Risk adjustment based on the multilevel model attenuated but did not eliminate these differences, and the Southwest China hospital remained the highest-burden site after standardization to the overall patient covariate distribution (Table 6).

**Table 6:** Hospital-level crude and adjusted prevalence of unmet needs.

Hospital (region)	*n*	Crude prevalence *n* (%)	Crude 95% CI (%)	Adjusted prevalence (%)	Adjusted 95% CI (%)
Hospital E (East China)	438	323 (73.7)	69.4–77.6	77	73.6–80.2
Hospital M (Middle China)	411	322 (78.3)	74.1–82.1	77.7	74.0–81.0
Hospital S (Southwest China)	390	324 (83.1)	79.0–86.5	81.6	78.1–84.7

## 4 Discussion

In this three-hospital multicenter cohort of Chinese head and neck cancer survivors within 3 to 24 months after treatment, unmet supportive care needs were common. The highest burdens were observed in health system and information and psychological domains, with additional head and neck-specific needs concentrated in dry mouth or sticky saliva, swallowing difficulties, nutrition information, and hearing concerns. Lower UW-QOL composite score, socioeconomic disadvantage, advanced stage, multimorbidity, and shorter time from treatment completion were associated with higher odds of unmet need. Surgery-based treatment was associated with lower odds than radiotherapy-based treatment, while hospital differences remained after adjustment but should be interpreted cautiously because only three centers were included.

The overall prevalence of moderate-to-high unmet supportive care needs in our cohort was high at 78.2% within 3–24 months after treatment, exceeding the 50% overall moderate-high unmet need reported in a large multinational cohort of head and neck cancer survivors surveyed more than 5 years after diagnosis using SCNS-SF34 and SCNS-HNC (19). Several methodological differences likely contribute to this discrepancy. First, our survivorship window (3–24 months post-treatment) captures early to mid-survivorship when needs are typically higher, whereas Jansen et al. restricted inclusion to long-term survivors beyond 5 years, a period when domain-level unmet needs (e.g., psychological 25%, physical and daily living 22%) remain substantial but are generally lower than earlier time points (19). Second, our clinic-based sampling may overrepresent survivors with more complex late effects compared with more heterogeneous multicenter or registry-based samples. Third, prior head and neck cohorts using SCNS-SF34 have generally involved mixed head and neck cancer sites rather than nasopharyngeal cancer-dominant populations (19, 39); the heavier radiotherapy-related toxicity profile typical of nasopharyngeal cancer may magnify supportive care needs, particularly in physical, lifestyle, and head and neck-specific domains. Finally, we operationalized “unmet need” at the person level as the presence of at least one item rated moderate or high, yielding a deliberately sensitive prevalence estimate, whereas Jansen et al. dichotomized unmet need at the domain level and then across domains (19), and earlier work in head and neck survivorship at approximately 2 years post-treatment has reported substantially lower domain-level moderate-high unmet needs (e.g., 17% physical/daily living, 14% psychological, 8% sexuality, 5% lifestyle) using domain thresholds rather than an “any-item” criterion (19).

In our early survivorship cohort, health system and information (34.2%) and psychological needs (31.2%) were the most prevalent domains, with substantial physical and daily living needs (28.7%), a rank order broadly consistent with SCNS-based oncology literature but with some important nuances. In mixed-cancer, largely clinic-based samples from China and Jordan, health system/information and psychological domains also scored highest, ahead of physical and daily living needs, mirroring our pattern and underscoring how informational gaps and emotional adjustment dominate soon after treatment in hospital-centered models of care (17, 40). In contrast, long-term multinational head and neck cohorts beyond 5 years post-diagnosis show psychological and physical/daily living needs as leading domains, with informational domains often omitted or lower priority, suggesting a shift from system-navigation issues toward enduring emotional and functional sequelae over time and in more mature survivorship systems (19, 41).

In keeping with radiotherapy-heavy nasopharyngeal and head and neck cancer survivorship literature, xerostomia-related problems were the leading unmet head and neck-specific need in our cohort (dry mouth or sticky saliva 54.6%), mirroring multinational SCNS-HNC data in which dry mouth/sticky mucus is also the single most prevalent item (24%) years after treatment (19). Swallowing difficulties (36.8%) and the need for nutrition or diet information (31.7%) similarly align with reports that dysphagia and weight/nutrition problems are among the most common long-term head and neck-specific unmet needs, though often at lower absolute prevalence (chewing/swallowing 20%, weight problems 12%, information on nutrition 7%) (19). This gradient is consistent with high dysphagia and nutrition-impact symptom burdens described in radiotherapy cohorts and qualitative work on chronic NIS (42–44). By contrast, hearing problems (27.3%) were more prominent in our nasopharyngeal cancer-dominant sample than in mixed-site cohorts, where hearing appears as a mid-ranked HNC-specific need (~11%) rather than a top-tier survivorship gap (19).

Our UW-QOL v4 profile, with saliva (55.1), taste (63.6), and swallowing (69.1) as lowest domains, aligns closely with contemporary HNC and NPC survivorship cohorts, where post-radiotherapy impairments in saliva and taste are among the most affected functions and often worse in advanced or multimodality treatment groups (26, 27, 36, 45). Longitudinal data show that most global and functional scores improve after the first year, but saliva and taste frequently plateau at persistently low levels, while swallowing improves more clearly yet rarely returns to baseline, especially after chemoradiotherapy or combined-modality regimens (26, 27, 45–47). This contrasts with relatively preserved pain and activity domains in both early and longer-term follow-up, and with higher function in single-modality or surgery-only cohorts (26, 36, 45).

Lower UW-QOL composite scores showing higher odds of unmet needs per 10-point decrement (aOR 1.61) are consistent with broader cancer and HNC literature, where poorer global QoL and heavier symptom burden strongly predict greater unmet needs even after multivariable adjustment (19, 28, 40, 48, 49). Population-based and longitudinal cohorts demonstrate that this association is robust to sociodemographic, treatment, and time-since-treatment factors, with physical/daily living and psychological QoL decrements particularly linked to higher needs (19, 28, 40, 49). Conceptually parallel models in Chinese and European samples show that unmet needs mediate or closely track the impact of somatic symptom load and functional limitations on overall QoL (28, 50, 51). However, most prior work has operated at domain or global QoL level, with relatively few studies dissecting salivary, swallowing, or mood/anxiety subdomains as stronger drivers than composite indices (19, 27, 36, 50).

Because supportive care needs and quality of life are conceptually adjacent constructs, the strength of the UW-QOL association should not be read as evidence of an entirely independent causal pathway. Instead, the coefficient likely captures a broader survivorship-burden phenotype in which symptom severity, functional limitation, and unmet supportive needs co-occur.

Socioeconomic gradients in unmet needs in our cohort, with excess risk among rural residents (aOR 1.25) and low-income survivors (aOR 1.45) and protection from higher education (aOR 0.66), mirror international survivorship evidence linking financial toxicity and deprivation to poorer access, greater distress, and residual disparities despite guideline-based care (52–54). Rurality amplifies travel time, transport costs, and reliance on centralized oncology and rehabilitation services, heightening informational, logistical, and financial needs even when psychosocial outcomes appear similar overall (7, 55–57). In China, fragmented insurance schemes, urban–rural resource gaps, and lower health literacy in disadvantaged groups further constrain navigation of follow-up and rehabilitation (58, 59). Health literacy and survivorship care planning interventions partly mitigate these gradients but rarely eliminate needs in structurally disadvantaged populations (60, 61).

Clinical burden gradients in our cohort, with higher unmet needs among survivors with stage III-IV disease (aOR 1.25) and multimorbidity (aOR 1.83) and lower needs at 13–24 versus 3–6 months post-treatment (aOR 0.65), broadly align with survivorship studies linking advanced stage, greater treatment intensity, comorbidities, and shorter time since diagnosis to elevated supportive care needs across cancer types (17, 19, 62). Advanced stage frequently proxies multimodality regimens and late-effect burden, with higher physical, psychological, informational, and financial needs in late-stage or heavily treated survivors, independent of sociodemographic factors (17, 19). However, time-course patterns are mixed: while many breast and mixed-cancer cohorts show highest needs in early survivorship that attenuate over years (20, 62), colorectal reviews report persistent or even higher unmet needs in extended survivorship, especially physical domains (54, 63). The data suggest front-loaded but often long-tailed need trajectories.

Treatment-related differences in unmet needs in our cohort, with lower odds after surgery-based treatment versus radiotherapy with or without chemotherapy (aOR 0.72), are consistent with head and neck survivorship data showing more pronounced long-term symptom burden and supportive care needs after (chemo)radiotherapy and multimodality regimens than after surgery alone (19, 26, 64). Radiotherapy-attributable late effects, xerostomia, dysphagia, trismus, sticky saliva, fatigue, and pain, frequently persist for years and cluster with higher informational, rehabilitative, and psychosocial needs (65–67). In NPC, where high-dose skull-base irradiation is standard, longitudinal studies similarly document durable QOL decrements, particularly in swallowing and salivary domains, driving ongoing rehabilitation requirements (13, 66, 68). However, once symptom and function measures are included, NPC status itself typically loses independent prognostic value, with unmet needs more tightly linked to treatment intensity and late-effect severity than to primary site per se (19, 26, 64).

Our observed inter-hospital spread in crude unmet needs (73.7–83.1%), with risk-adjusted prevalence still highest in the Southwest center, parallels multicountry survivorship work showing persistent between-system and between-region variation after adjustment for case mix and clinical factors (17, 19, 40). In long-term head and neck survivors, differences in overall, physical, and HNC-specific needs by European subregion and health-system type remained after controlling for stage, treatment, performance status, and comorbidity, suggesting that configuration and accessibility of survivorship services contribute meaningfully to need profiles (19). Explanations proposed include differential availability and integration of swallowing rehabilitation, dental surveillance, dietetics, and psychosocial oncology, as well as fragmented care pathways and travel distance barriers (1, 19, 65). Broader Asia-Pacific and global surveys similarly implicate regional socioeconomic context and resource constraints in sustaining higher unmet needs even after demographic and clinical adjustment (17, 40, 69). Even so, with only three hospitals, the observed between-hospital spread should be interpreted as hypothesis-generating rather than definitive evidence of system-level effects. The fixed-effect sensitivity analysis is useful for judging whether patient-level findings are stable, but it cannot fully resolve uncertainty around center-level inference.

### 4.1 Limitations and implications

There are several limitations. The study used a cross-sectional, clinic-based consecutive sample from tertiary follow-up services, so the results may not generalize to community-based or surgery-dominant survivor populations, and temporal ordering cannot be established. Outcomes were self-reported over short recall windows and may therefore be affected by recall, reporting, and social-desirability bias. The cohort was NPC-heavy and predominantly radiotherapy-treated. Hospital clustering was modeled with only three centers, so hospital-level variance estimates and adjusted between-hospital differences should be treated as descriptive. In addition, the use of an any-item SCNS-SF34 definition is intentionally sensitive for screening and may produce higher patient-level prevalence than stricter domain-based definitions. Although reliability and diagnostic checks were acceptable in this draft, all supplementary quantitative details should be confirmed against the analytic dataset before resubmission.

## 5 Conclusion

Unmet supportive care needs are common among Chinese head and neck cancer survivors during early survivorship, especially in information, psychological, salivary, swallowing, and nutrition-related domains. Poorer head and neck-related quality of life, socioeconomic disadvantage, and greater clinical burden are associated with higher unmet need. Our findings support routine needs assessment during follow-up and suggest that targeted supportive care pathways may be particularly relevant for socioeconomically vulnerable survivors, while hospital-level differences should be interpreted cautiously.

## Data Availability

The raw data supporting the conclusions of this article will be made available by the authors, without undue reservation.
